# Association of blood pressure and renal outcome in patients with chronic kidney disease; a post hoc analysis of FROM-J study

**DOI:** 10.1038/s41598-021-94467-z

**Published:** 2021-07-22

**Authors:** Mariko Tsuchida-Nishiwaki, Haruhito A. Uchida, Hidemi Takeuchi, Noriyuki Nishiwaki, Yohei Maeshima, Chie Saito, Hitoshi Sugiyama, Jun Wada, Ichiei Narita, Tsuyoshi Watanabe, Seiichi Matsuo, Hirofumi Makino, Akira Hishida, Kunihiro Yamagata

**Affiliations:** 1 Department of Nephrology, Rheumatology, Endocrinology and Metabolism, Okayama University Graduate School of Medicine, Dentistry, and Pharmaceutical Science, Okayama, Japan; 2 Department of Chronic Kidney Disease and Cardiovascular Disease, Dentistry, and Pharmaceutical Science, Okayama University Graduate School of Medicine, Dentistry, and Pharmaceutical Science, Okayama, Japan; 3 Department of Gastroenterological Surgery Transplant and Surgical Oncology, Okayama University Graduate School of Medicine, Dentistry, and Pharmaceutical Science, Okayama, Japan; 4 University of Hyogo, Hyogo, Japan; 5 Department of Nephrology, Faculty of Medicine, University of Tsukuba, Tsukuba, Ibaraki Japan; 6 Department of Human Resource Development of Dialysis Therapy for Kidney Disease, Okayama University Graduate School of Medicine, Dentistry, and Pharmaceutical Science, Okayama, Japan; 7 Division of Clinical Nephrology and Rheumatology, Niigata University Graduate School of Medical and Dental Science, Niigata, Japan; 8 Tokyo-Kita Medical Center, Kita-ward, Tokyo, Japan; 9 Nagoya University, Nagoya, Aichi Japan; 10 Okayama University, Okayama, Japan; 11 Yaizu City Hospital, Shizuoka, Japan; 12 Department of Medicine and Clinical Science, Okayama University Graduate School of Medicine, Dentistry, and Pharmaceutical Science, 2-5-1 Shikata-cho, Kita-ku, Okayama, 700-8558 Japan

**Keywords:** Nephrology, Hypertension

## Abstract

It is well-known that hypertension exacerbates chronic kidney disease (CKD) progression, however, the optimal target blood pressure (BP) level in patients with CKD remains unclear. This study aimed to assess the optimal BP level for preventing CKD progression. The risk of renal outcome among different BP categories at baseline as well as 1 year after, were evaluated using individual CKD patient data aged between 40 and 74 years from FROM-J [Frontier of Renal Outcome Modifications in Japan] study. The renal outcome was defined as ≥ 40% reduction in estimated glomerular filtration rate to < 60 mL/min/1.73 m^2^, or a diagnosis of end stage renal disease. Regarding baseline BP, the group of systolic BP (SBP) 120–129 mmHg had the lowest risk of the renal outcome, which increased more than 60% in SBP ≥ 130 mmHg group. A significant increase in the renal outcome was found only in the group of diastolic BP ≥ 90 mmHg. The group of BP < 130/80 mmHg had a benefit for lowering the risk regardless of the presence of proteinuria, and it significantly reduced the risk in patients with proteinuria. Achieving SBP level < 130 mmHg after one year resulted in a 42% risk reduction in patients with SBP level ≥ 130 mmHg at baseline. Targeting SBP level < 130 mmHg would be associated with the preferable renal outcome.

Clinical Trial Registration-URL: https://www.umin.ac.jp/ctr/. Unique identifier: UMIN000001159 (16/05/2008).

## Introduction

Chronic kidney disease (CKD) is a worldwide health problem, which has a high global prevalence estimated between 11 and 13%, and also has similar prevalence among the Japanese population^1,2^. Additionally, in many countries, as well as in Japan, the prevalence of CKD has continued to rise^3^. CKD is an established risk factor for not only end stage renal disease (ESRD) but also cardiovascular disease (CVD) and all-cause mortality^4,5^. Given these facts, prevention of CKD progression is one of the top public health priorities.

Hypertension is present in approximately 80 to 85% of patients with CKD, which is much higher compared to patients in the general population^6–9^. In addition, blood pressure (BP) is strongly associated with kidney function and hypertension exacerbates CKD progression and incidence of ESRD, as well as the risk of CVD^10–13^. Several clinical studies have shown that BP reduction decreased those risks, however, the optimal target level of BP in patients with CKD remains unclear^14–17^. In fact, recent guidelines recommend to achieve lower BP goals, while the target BP level for patients with CKD varies depending on guidelines^18–20^. The evaluation on the relevance of the new guidelines is necessary for appropriate management of patients with CKD.

The FROM-J [Frontier of Renal Outcome Modifications in Japan] study, which investigated the effect of lifestyle modification on the outcome of patients with CKD, is one of the most extensive prospective trials of patients with CKD in Japan^21^. In the FROM-J study, all patients received treatment in accordance with the CKD clinical practice guideline of Japanese Society of Nephrology, which means that all enrolled patients were treated to maintain the same BP level. Patients in the group A (standard intervention) were only instructed to follow the guideline^22^. On the other hand, patients in the group B (advanced intervention) received educational sessions from dieticians and a letter regarding ideal lifestyle for CKD, as well as a notification one week before the consultation^21^. In the FROM-J study, the association of BP levels and CKD progression has not been explored yet.

In the present study, we analyzed the relationship between patients’ BP levels and renal outcome. We aimed to examine the most preferable association of BP level and renal outcome in the patients with CKD.

## Results

### Baseline characteristics of study patients

Of the 2100 patients included in these analyses, 975 (46.4%) were categorized as CKD stage 1 or 2 and 1125 (53.6%) were CKD stage 3 to 5, the mean systolic BP (SBP) (± SD) was 137 ± 16 mmHg and that of diastolic BP (DBP) was 79 ± 11 mmHg, and the prevalence of hypertension was 90.8%. The baseline characteristics of patients are shown in Table 1 by SBP category and in Supplemental Table S1 by DBP category. A total of 350 patients (16.7%) experienced the renal outcome during a median follow-up period of 5.1 years (interquartile range [IQR], 5.1–5.2 years).

**Table 1:** Baseline characteristics of patients stratified by SBP categories.

Characteristics	Overall n = 2100	Baseline SBP					
		< 120 n = 229	120–129 n = 348	130–139 n = 604	140–149 n = 445	150–159 n = 253	160 ≤ n = 191
Male sex, %	71.4	72.9	74.3	69.5	69.7	73.5	71.2
Age, year	62.5 ± 8.3	62.4 ± 8.7	61.9 ± 8.8	62.7 ± 8.2	62.3 ± 8.0	62.9 ± 7.7	63.0 ± 8.4
BMI, kg/m^2^	25.7 ± 3.8	25.4 ± 3.7	25.6 ± 3.8	25.5 ± 3.7	25.8 ± 4.0	26.1 ± 3.7	25.8 ± 4.1
Abdominal girth, cm	90.0 ± 9.9	87.4 ± 10.7	89.0 ± 9.8	90.2 ± 9.7	91.2 ± 9.8	90.6 ± 9.1	91.1 ± 10.4
Current smoking, %	22.5	19.4	24.5	21.7	22.5	24.2	23.0
Diabetes mellitus, %	61.1	58.1	58.5	59.0	61.0	68.4	67.0
Hypertension, %	90.8	77.7	87.5	91.0	95.1	96.1	95.8
Dyslipidemia, %	69.3	66.8	69.5	69.3	71.2	72.3	63.4
Hyperuricemia, %	38.8	39.3	40.3	40.4	35.6	35.2	42.4
**Medication**							
Anti-hypertensive, %	86.1	73,8	81.8	85.9	89.4	93.3	93.2
Use of RAS-I, %	72.8	65.1	70.9	70.5	74.6	79.8	79.6
**Laboratory values**							
Positive proteinuria, %^a^	80.6	71.6	73.7	80.3	85.4	88.5	85.3
Proteinuria, median (IQR), g/gCr	0.22 (0.06–0.71)	0.16 (0.05–0.39)	0.19 (0.06–0.55)	0.23 (0.06–0.72)	0.26 (0.07–0.87)	0.28 (0.10–0.85)	0.45 (0.08–1.36)
Total protein, g/dL	7.2 ± 0.5	7.2 ± 0.5	7.2 ± 0.5	7.3 ± 0.5	7.3 ± 0.5	7.1 ± 0.5	7.2 ± 0.5
Albumin, g/dL	4.3 ± 0.4	4.2 ± 0.3	4.3 ± 0.3	4.3 ± 0.4	4.3 ± 0.4	4.2 ± 0.4	4.2 ± 0.4
Hemoglobin, g/dL	13.8 ± 1.8	13.6 ± 1.8	13.9 ± 1.8	13.8 ± 1.8	13.9 ± 1.7	13.8 ± 1.9	13.6 ± 2.1
BUN, mg/dL	19.6 ± 8.0	20.2 ± 8.0	18.9 ± 7.2	19.5 ± 8.0	19.4 ± 8.1	19.5 ± 8.4	20.7 ± 8.7
Creatinine, mg/dL	1.07 ± 0.49	1.10 ± 0.44	1.05 ± 0.48	1.07 ± 0.48	1.05 ± 0.49	1.10 ± 0.57	1.16 ± 0.53
eGFR, ml/min/1.73 m^2^	59.1 ± 21.9	57.0 ± 21.2	60.3 ± 21.1	59.0 ± 22.1	60.8 ± 22.2	59.1 ± 21.7	55.7 ± 22.7
HbA1c, %	6.2 ± 1.2	6.0 ± 1.1	6.1 ± 1.2	6.1 ± 1.1	6.2 ± 1.3	6.3 ± 1.2	6.3 ± 1.1
Uric acid, mg/dL	6.2 ± 1.2	6.2 ± 1.8	6.2 ± 1.5	6.1 ± 1.5	6.1 ± 1.6	6.0 ± 1.5	6.5 ± 1.8
TC, mg/dL	198 ± 35	193 ± 34	195 ± 34	197 ± 37	201 ± 37	195 ± 357	203 ± 33
HDL-C, mg/dL	54 ± 16	55 ± 16	53 ± 15	54 ± 15	56 ± 17	53 ± 14	54 ± 18
Non-HDL-C, mg/dL	147 ± 37	139 ± 34	145 ± 36	147 ± 37	149 ± 39	145 ± 36	153 ± 39
Triglyceride, mg/dL	173 ± 140	151 ± 98	170 ± 131	172 ± 118	176 ± 171	176 ± 136	202 ± 176

Unless otherwise specified, data are presented as the mean ± SD.

*SBP* systolic blood pressure, *BMI* body mass index, *CKD* chronic kidney disease, *DBP* diastolic blood pressure, *RAS-I* renin angiotensin system inhibitor, *IQR* interquartile range, *BUN* blood urea nitrogen, *eGFR* estimated glomerular filtration rate, *HbA1c* hemoglobin A1c, *TC* total cholesterol, *HDL-C* high density lipoprotein cholesterol.

^a^Dipstick positive proteinuria of ± or higher.

### The association of BP levels at baseline and the renal outcome

**Figure 1:**
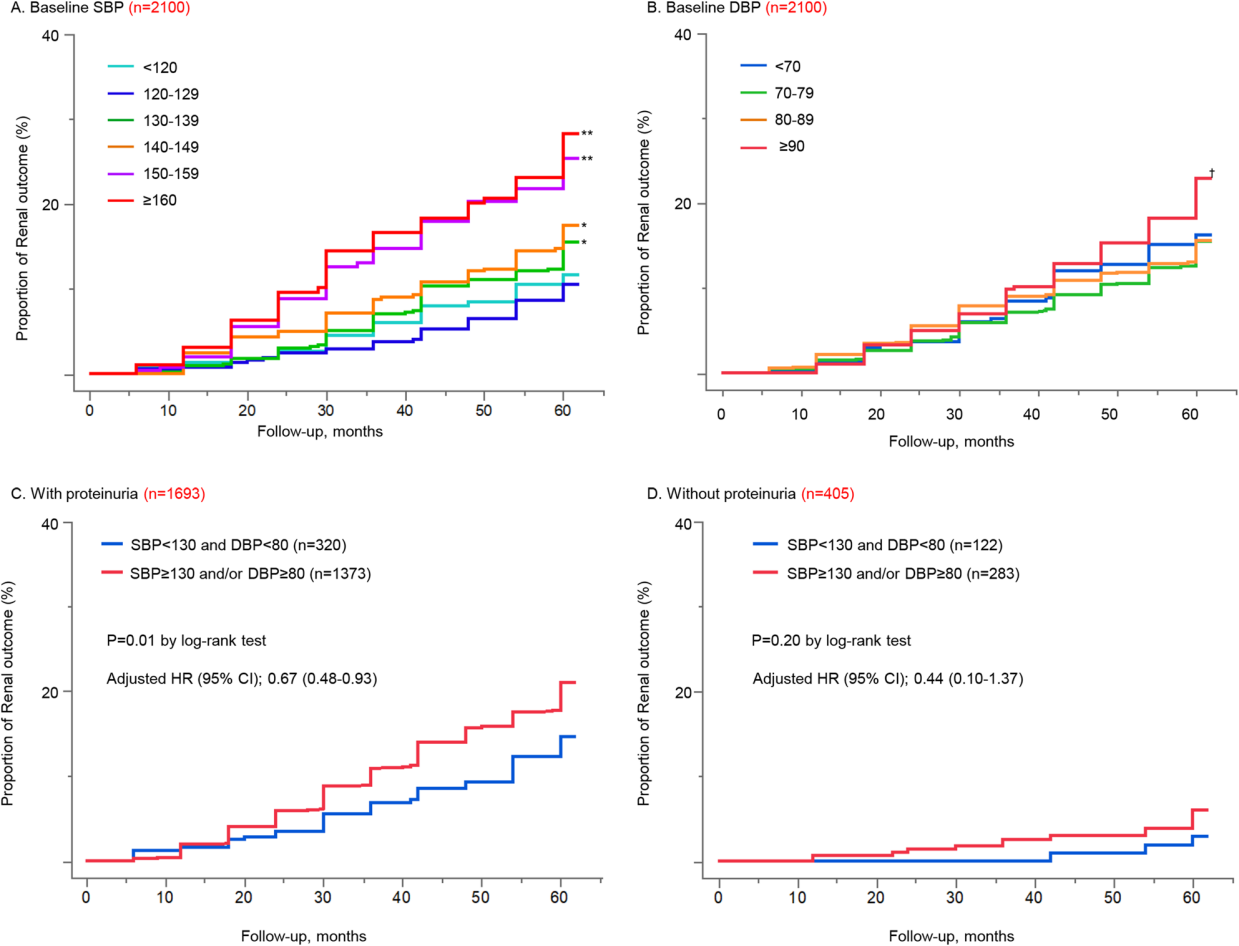
Kaplan–Meier analyses for renal outcome based on baseline SBP and DBP levels **(A,B)**, and stratified by the presence or absence of baseline proteinuria** (C,D).** **(A)** Analyses of baseline SBP levels (6 groups); The SBP 120–129 mmHg group had the lowest risk of the renal outcome. **(B)** Analyses of baseline DBP levels (4 groups); the risk of renal outcome significantly increased only in the DBP ≥ 90 mmHg group compared to the DBP 70–79 mmHg group. *p < 0.05 vs SBP 120–129 mmHg (Log-rank), **p < 0.001 vs SBP 120–129 mmHg (Log-rank), ^†^p < 0.05 vs DBP 70–79 mmHg (Log-rank). **(C)** Patients with proteinuria; proteinuria was defined as ≥  ± by dipstick test. The baseline BP < 130/80 mmHg group significantly reduced the risk of the renal outcome compared to the other. **(D)** Patients without proteinuria; No significant difference was found in renal outcome. *SBP* systolic blood pressure, *DBP* diastolic blood pressure, *BP* blood pressure, *HR* hazard ratio. Hazard ratios have been adjusted for nine prespecified baseline factors (age, sex, body mass index, smoking, presence of diabetes mellitus, presence of dyslipidemia, presence of hyperuricemia, use of anti-hypertensive medication and intervention arm).

A strong association between baseline SBP levels and the renal outcome was identified. The SBP 120–129 mmHg group had the lowest risk of the renal outcome, which increased by more than 60% in the SBP 130–139 mmHg, 140–149 mmHg, 150–159 mmHg and SBP ≥ 160 mmHg groups. (Fig. 1 and Table 2). The association between baseline DBP levels and the renal outcome was weaker than that of SBP. The significant increase was only observed in the DBP ≥ 90 mmHg group compared with the DBP 70–79 mmHg group (Fig. 1 and Table 2). The increased risk of the renal outcome at higher SBP levels was even greater in patients with an estimated glomerular filtration rate (eGFR) < 60 ml/min/1.73 m^2^. The SBP 120–129 mmHg group had the lowest risk, which increased more than two folds in the SBP ≥ 130 mmHg groups. (see Supplemental Figure S1 and Supplemental Table S2).

**Table 2:** Risk of renal outcomes based on baseline SBP and DBP levels.

(A) Baseline SBP			
Baseline SBP	Events, %	Adjusted HR (95% CI)	*P* value
< 120	10.5	1.29 (0.75–2.18)	0.345
120–129	9.5	1 [reference]	
130–139	13.7	1.60 (1.08–2.41)	0.018
140–149	16.0	1.77 (1.18–2.71)	0.005
150–159	22.9	2.73 (1.77–4.26)	< 0.001
≥ 160	26.2	2.88 (1.86–4.52)	< 0.001

(B) Baseline DBP			
Baseline DBP	Events, %	Adjusted HR (95% CI)	*P* value
< 70	15.1	1.01 (0.70–1.44)	0.961
70–79	13.9	1 [reference]	
80–89	14.2	1.08 (0.81–1.44)	0.588
≥ 90	20.6	1.72 (1.24–2.37)	0.001

(C) Risk ratios of renal outcomes based on baseline BP levels			
Risk ratio (95% CI)	DBP < 70	70 ≤ DBP < 90	DBP ≥ 90
SBP < 130	1.28 (0.77–2.12)	1 [reference]	1.39 (0.46–4.19)
130 ≤ SBP < 150	2.29 (1.38–3.81)	1.51 (1.06–2.16)	1.85 (1.16–2.95)
SBP ≥ 150	2.30 (1.06–5.00)	2.68 (1.82–3.95)	2.79 (1.87–4.16)

Hazard ratios have been adjusted for nine prespecified baseline factors (age, sex, body mass index, smoking, presence of diabetes mellitus, presence of dyslipidemia, presence of hyperuricemia, use of anti-hypertensive medication and intervention arm).

*SBP* systolic blood pressure, *DBP* diastolic blood pressure, *HR* hazard ratio, *CI* confidence interval.

To examine the combined effects of various levels of SBP and DBP on the renal outcome, we classified baseline BP levels into nine groups, in which SBP was classified into three; < 130 mmHg, 130–149 mmHg and ≥ 150 mmHg, and DBP was into three; < 70 mmHg, 70–89 mmHg and ≥ 90 mmHg. We then calculated the risk ratios of the renal outcome for each group. The BP < 130/70–89 mmHg group had the lowest frequency of the renal outcome, then it was used as a reference to calculate the risk ratios. No significant increase was observed in the BP < 130/ < 70 mmHg and < 130/ ≥ 90 mmHg groups. On the contrary, those with an SBP ≥ 130 mmHg had a significantly increased risk. When the DBP levels were the same, the risk ratios increased as the SBP level increased, while the risk ratios did not increase as the DBP level raised when the SBP levels were same (Table 2). The risk ratio results consisted of six SBP groups and four DBP groups is shown in Supplemental Table S2.

For estimating varying impact of managing BP on the renal outcome due to the presence of proteinuria, the difference in the renal outcome between the two baseline BP levels, which were BP < 130/80 mmHg and SBP ≥ 130 mmHg and/or DBP ≥ 80 mmHg, was compared in patients with and without proteinuria, respectively. Proteinuria was defined as baseline proteinuria ± or more by dipstick test. Two patients were excluded in this subgroup analysis because of the lack of proteinuria measurement at baseline. The association of baseline BP levels with renal outcome differed according to the presence of baseline proteinuria levels (Fig. 1). Among patients with proteinuria, the baseline BP < 130/80 mmHg group significantly reduced the risk of the renal outcome. We also found that the risk of renal outcome tended to reduce in those with a baseline BP < 130/80 mmHg among patients without proteinuria, although the difference between the two baseline BP levels did not reach the statistical significance.

### The association BP levels after 1 year with renal outcome

We investigated the association between the renal outcome and BP levels at baseline and 1 year later. A total of 56 patients were excluded because of missing measurement of the BP level of 1 year later or the renal outcome reached within 12 months. The achiever was defined as patients who attained the target SBP or DBP levels at both baseline and after 1 year: the late failure as patients who attained the target SBP or DBP at baseline but not after 1 year: the late achiever as patients who did not attain the target SBP or DBP at baseline but achieved after 1 year: and the failure as patients who did not attain the target SBP or DBP both at baseline and after one year.

**Figure 2:**
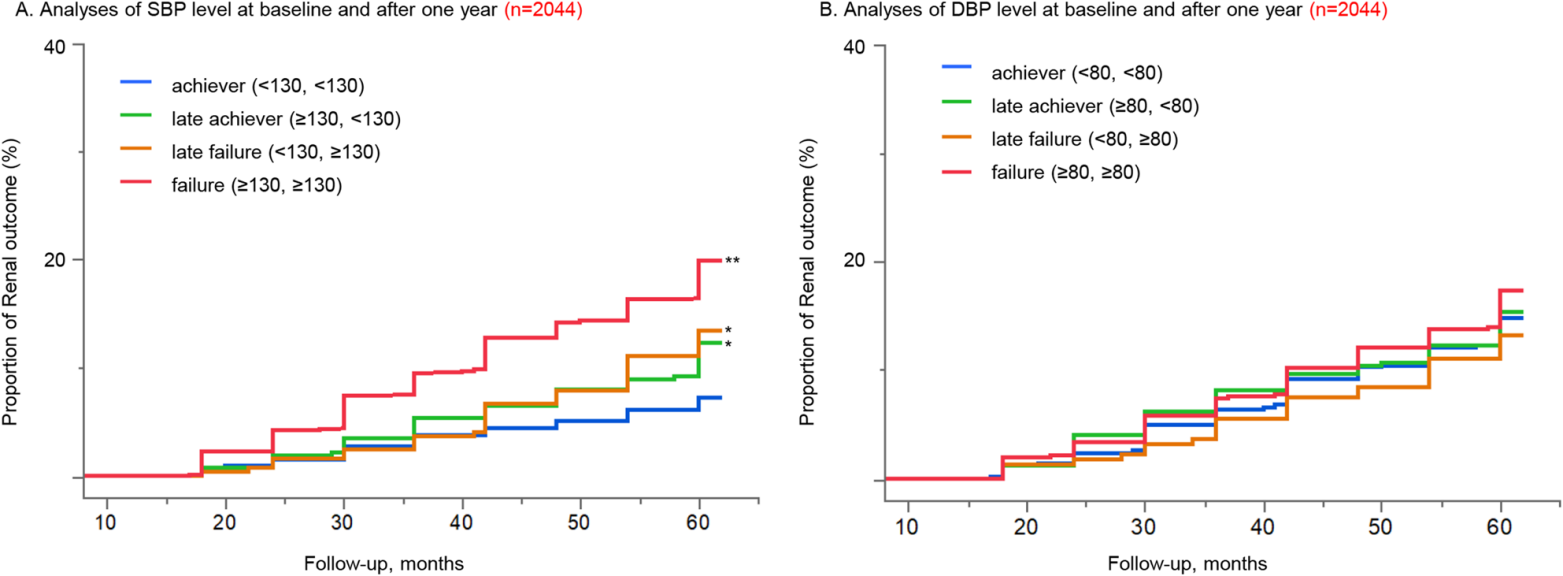
Kaplan–Meier analyses of renal outcome based on management of BP levels after 1 year (n = 2044). Patients were divided into 4 groups according to management of SBP and DBP levels, respectively, at baseline and after 1 year. **(A)** Analyses of SBP level at baseline and after 1 year; the failure and the late achiever had significant higher risk of renal outcome compared to the achiever. (B) Analyses of DBP level at baseline and after 1 year; no significant difference was found among groups. *p < 0.05 vs achiever (Log-rank), **p < 0.001 vs achiever (Log-rank). *BP* blood pressure.

A significant association between SBP change and the risk of renal outcome was found. The failure significantly increased the risk of renal outcome by more than 3.1 times in comparison with the achiever (Fig. 2 and Table 3). The difference between the late failure and the achiever increased over the follow-up period, and the late failure reached an increase of more than 80% over the achiever. The late achiever also resulted in a significant increase compared to the achiever, however, it reduced the risk by 42% compared to the failure (HR, 0.58; 95% CI, 0.41–0.80). No significant difference in renal outcome risk was observed in the DBP management.

**Table 3:** Risk of renal outcomes based on management of blood pressure levels after one year (n = 2044).

(A) SBP at baseline and after 1 year				
Group	SBP		Adjusted HR (95% CI)	*P* value
	Baseline	After 1 year		
Achiever	< 130	< 130	1 [reference]	
Late achiever	≥ 130	< 130	1.80 (1.07–3.11)	0.027
Late failure	< 130	≥ 130	1.81 (1.04–3.22)	0.036
Failure	≥ 130	≥ 130	3.11 (2.02–5.05)	< 0.001

(B) DBP at baseline and after 1 year				
Group	DBP		Adjusted HR (95% CI)	*P* value
	Baseline	After 1 year		
Achiever	< 80	< 80	1 [reference]	
Late achiever	≥ 80	< 80	1.07 (0.77–1.49)	0.669
Late failure	< 80	≥ 80	0.91 (0.56–1.41)	0.681
Failure	≥ 80	≥ 80	1.32 (0.98–1.78)	0.068

Hazard ratios have been adjusted for nine prespecified baseline factors (age, sex, body mass index, smoking, presence of diabetes mellitus, presence of dyslipidemia, presence of hyperuricemia, use of anti-hypertensive medication and intervention arm).

*SBP* systolic blood pressure, *DBP* diastolic blood pressure, *HR* hazard ratio, *CI* confidence interval.

A subgroup analysis of 541 patients with severe CKD with eGFR < 45 ml/min/1.73 m^2^ also demonstrated these trends (Supplemental Figure S2 and Supplemental Table S3). This analysis showed that the late achiever provided a 63% reduction in the risk of renal outcomes compared with the failure. (HR of the late achiever versus the failure, 0.37; 95% CI, 0.21–0.60).

### Comparison of the Group A with B

**Figure 3:**
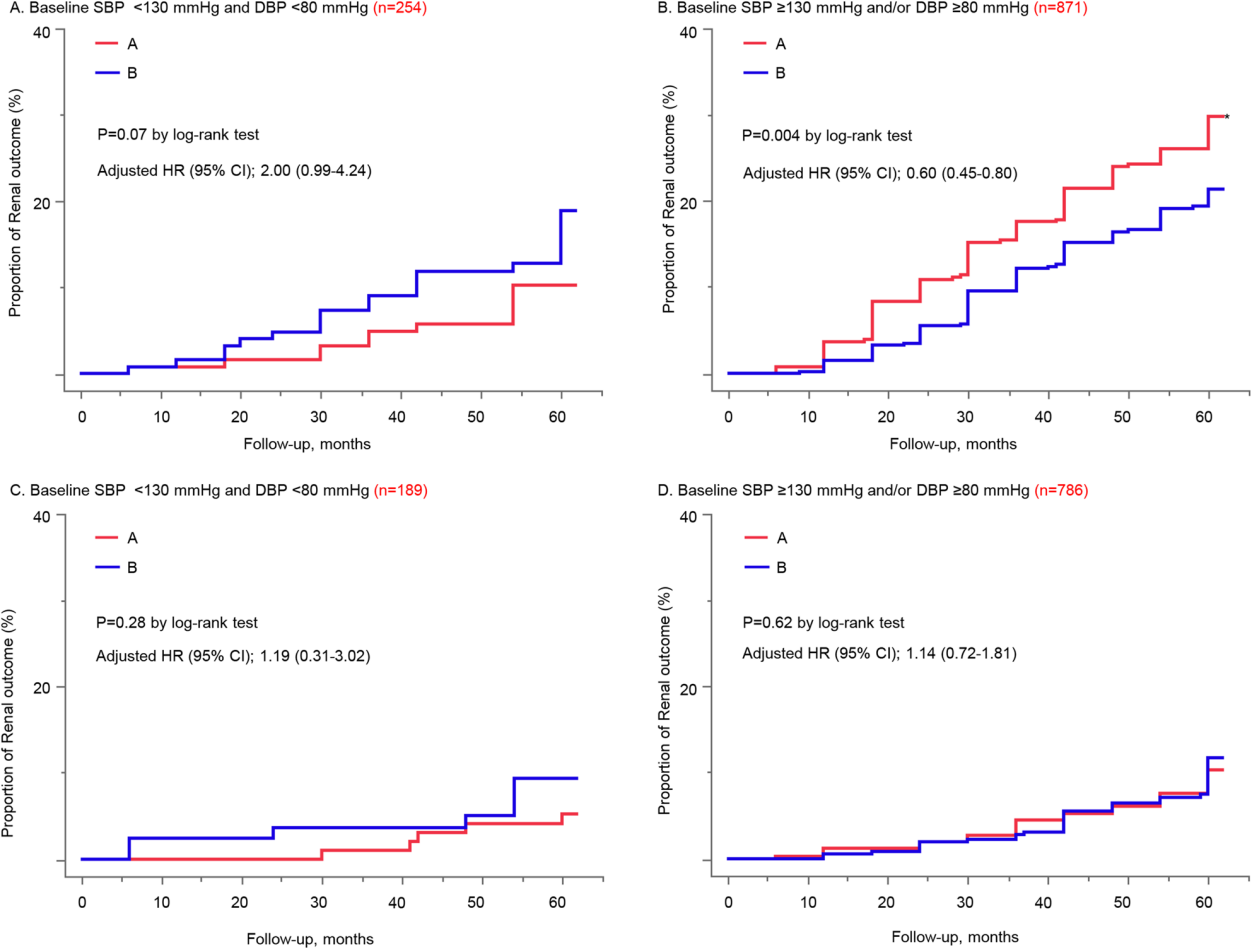
Kaplan–Meier analyses of renal outcome between the group A and B, stratified by baseline BP levels in patients with eGFR < 60 ml/min/1.73 m^2^ **(A,B) **and ≥ 60 ml/min/1.73 m^2^** (C,D).** The difference in the effect on renal outcome between the group A and B was compared in the patients with eGFR < 60 ml/min/1.73 m^2^ **(A,B)**. Patients in group A (standard intervention) were simply instructed to follow the medical guidelines of the Japanese Society of Nephrology. On the other hand, patients in group B (advanced intervention) received an educational session by a dietitian and a letter regarding ideal lifestyle for CKD and were notified 1 week prior to the consultation. **(A)** Baseline SBP < 130 mmHg and DBP < 80 mmHg; No significant difference was observed. **(B)** The group B significantly reduced renal outcome compared to the group A. No significant difference was observed in patients with eGFR ≥ 60 ml/min/1.73 m^2^ **(C,D)**. *BP* blood pressure, *SBP* systolic blood pressure, *DBP* diastolic blood pressure, *eGFR* estimated glomerular filtration rate.

Furthermore, we compared the group A and B in accordance with their BP levels on renal outcome in patients with baseline eGFR < 60 ml/min/1.73 m^2^ (n = 1125) and those with baseline eGFR ≥ 60 ml/min/1.73 m^2^ (n = 975), respectively. Analysis of patients with eGFR < 60 ml/min/1.73 m^2^ showed that the group B had a significant reduction in patients with a baseline SBP ≥ 130 mmHg and/or DBP ≥ 80 mmHg, despite no significant difference in those with baseline BP < 130/80 mmHg. We found no difference of renal outcome between the group A and B in patients with eGFR ≥ 60 ml/min/1.73 m^2^ (Fig. 3). The proportion of patients with a BP level < 130/80 mmHg after 1 year did not differ between group A (20.7%) and B (22.6%) among patients with eGFR < 60 ml/min/1.73 m^2^ whose baseline SBP ≥ 130 mmHg and/or DBP ≥ 80 mmHg (P = 0.51 by Chi-squared test).

## Discussion

In the present study, we found that baseline SBP level of ≥ 130 mmHg and DBP level of ≥ 90 mmHg was associated with the increase of the risk of the renal outcome in patients with CKD. The association of baseline BP levels with the renal outcome varied by baseline proteinuria level. Among patients with proteinuria, the higher baseline BP levels significantly increased the risk of the renal outcome. Second, the renal outcome was also strongly associated with the SBP level after one year. In fact, the late achiever had a 42% reduction of the risk compared to the failure. Third, among the patients with eGFR < 60 ml/min/1.73 m^2^ who did not meet the BP level of < 130/80 mmHg at baseline, the group B showed a significant association of lowering risk in renal outcome compared to the group A.

A few observational studies have shown that SBP level was independently associated with the presence of CKD and patients with baseline SBP level of ≥ 130 mmHg had increased risk of CKD progression^11,12,23–28^. Our analyses using baseline BP levels demonstrated that SBP level of ≥ 130 mmHg results in a significant association with increment of risk of the renal outcome: more than 60% increase in the risk among patients with all CKD stages. Moreover, the risk more than doubled in patients with eGFR < 60 ml/min/1.73 m^2^. The SBP 120–129 mmHg group had a lower risk of the renal outcome than the SBP < 120 mmHg group, although there was no significant difference. Thus, it is likely concluded that the optimal SBP level for better renal outcome in patients with CKD could be < 130 mmHg, rather than < 120 mmHg. Regarding DBP, as prior studies have shown, baseline DBP levels had less association with the renal outcome than baseline SBP levels^23,29^. The significantly increased risk of the renal outcome in the DBP ≥ 90 mmHg group suggests that DBP level < 90 mmHg may be better on renal outcome. Given the minor difference between the DBP 70–79 mmHg and 80–89 mmHg groups at baseline and among the groups categorized by DBP levels after one year, it is difficult to conclude that DBP level should be controlled at < 80 mmHg. Since decreased DBP level sometimes results from stiffening of the aorta, which is one of the risks of CKD progression, the benefits of DBP levels at < 80 mmHg may be obscured in patients with severe arterial stiffness^30^.

Higher degrees of urinary protein excretion are also associated with a more rapid progression of CKD^31,32^. In addition, the risk of CKD progression at higher BP levels is greater in the patients with proteinuria^31–33^. In previous studies, lowering BP level reduced the risk of CKD progression among patients with a high degree of proteinuria, despite the lack of a significant reduction among patients with small degree of proteinuria ^16,17,34^. In the present study, among patients with proteinuria, those with BP level of < 130/80 mmHg was significantly associated with risk reduction. However, among the patients without proteinuria, the risk reduction of those with a BP of < 130/80 mmHg did not reach significant difference but the tendency of decreased risk was found. These results suggest possible benefits for patients with CKD by controlling their BP at < 130/80 mmHg, regardless of presence or absence of proteinuria.

Varying target levels have been used for patients undergoing intensive BP control group and the results have been inconsistent^14–17,35–38^. The MDRD (the Modification of Diet in Renal Disease) and AASK (the African American Study of Kidney Disease and Hypertension) trials targeted mean arterial pressure to ≤ 92 mmHg (equivalent to approximately 125/75 mmHg)^36,37^. Both trials showed no significant benefit of intensive BP control on CKD progression, whereas, with extended follow-up, intensive BP control showed significant reduction of CKD progression in patients with elevated level of proteinuria. The SPRINT (the Systolic BP Intervention Trial) and ACCORD BP (Action to Control Cardiovascular Risk in Diabetes BP) trials targeted SBP to < 120 mmHg^15,35^. In these trials, no significant difference was observed between randomized groups in ≥ 50% decline in eGFR or ESRD. A few studies showed that intensive BP lowering had adverse effects on CKD progression^14,35^. Distinct from previous intervention trials, in the present study, all enrolled patients had the same BP target level of < 130/80 mmHg. The analyses on the management of SBP levels after one year suggests that achieving the target SBP level after one year could significantly retards CKD progression among patients with SBP level of ≥ 130 mmHg at baseline. In addition, the analyses imply the possibility that failing to meet the target SBP level after one year would exacerbate CKD progression even though it was achieved at baseline. In fact, the difference in the risk between the late failure and the achiever increased over the follow-up period, and the former had 80% increased risk compared to the latter. A post hoc analysis of SPRINT showed an increase in acute kidney injury events in the intensive treatment arm among the patients with eGFR < 45 ml/min/1.73 m^2^ ^39^. However, in the present subgroup analysis of patients with eGFR < 45 ml/min/1.73 m^2^, the late achiever was also associated with reduction of the risk of renal outcome compared to the failure. Moreover, the association of the reduction was even stronger than the analysis which included patients with eGFR ≥ 45 ml/min/1.73 m^2^. This fact may suggest that targeting a slightly moderate SBP level to < 130 mmHg, rather than to < 120 mmHg, would minimize adverse effects and maximize benefit for the renal outcome.

In the FROM-J study, BP levels during the study period and the anti-hypertensive medication were almost identical between the groups^21^. On the contrary, the average body mass index and hemoglobin A1c were significantly lower after one year in the group B compared to the group A. A significantly lower rate of absence from regular clinical visits and higher co-treatment rate with nephrologists were observed in the group B^21^. In the current study, the group B demonstrated a significantly reduced risk of renal outcome among the patients with baseline eGFR < 60 ml/min/1.73 m^2^ who did not meet the BP level of < 130/80 mmHg at baseline. The lack of a significant difference in BP levels between the group A and B suggests that treatment of comorbidities, such as diabetes and obesity, as well as lifestyle modifications, may have further benefits to improve renal prognosis in patients with reduced kidney function who do not achieve the target BP level.

Our analysis has several limitations. First, since this was a post hoc analysis of the FROM-J study, unknown risk factors, which may have affected the results, were not completely excluded. Second, patients aged ≥ 75 years were excluded from the present study; therefore, our findings are unable to be applied to patients aged ≥ 75 years. Third, the assessment for the potential adverse effects associated with SBP level of < 110 mmHg or DBP level of < 60 mmHg was not performed, due to the lack of the applicable patient’s number. Fourth, we lacked data on some potentially important unmeasured confounders such as duration of hypertension or BP levels before recruitment into the study. Fifth, the presence of proteinuria was based on the results of dip-stick test, which has the possibility of a false negative or false positive. However, dip stick test is simpler and less expensive and more commonly used than the quantitative test. It is therefore convenient for non-specialists and general practitioners (GPs).

In conclusion, the significant association of the SBP ≥ 130 mmHg and DBP ≥ 90 mmHg groups at baseline with the renal outcome was found. Taking the result of BP levels at baseline and after one year together, targeting the SBP level to < 130 mmHg would be preferable for preventing CKD progression. The current guidelines which recommend a target BP level of < 130/80 mmHg for CKD patients appears to be appropriate.

## Methods

### Design

The FROM-J study was a prospective cluster-randomized trial, and enrolled patients with CKD who were under consultation with GPs. Details of the study design have previously been published^21,40^. The study was a Central Institutional Review Board Program; the Committee on Ethics in Strategic Research of the Kidney Foundation, Japan, and the institutional review board at the University of Tsukuba examined and approved the implementation plans and their revision^21,40^. In brief, the local medical associations were randomly assigned to two intervention groups. All patients received treatment in accordance with the CKD clinical practice guideline of Japanese Society of Nephrology, and patients in the group A (standard intervention) were only instructed to follow the guideline^22^. On the other hand, patients in the group B (advanced intervention) received three additional interventions. Firstly, the group B patients received 30-min educational sessions from dieticians upon visiting their local GP offices every 3 months. Secondly, the group B patients received a CKD treatment report from the coordinating center bimonthly to learn about CKD and their ideal lifestyle to prevent progression. In addition, they received a notification 1 week before the consultation in order to prevent their withdrawal from treatment. Thirdly, the group B GPs received comments about their patients’ data, focusing on the gap between target and practice, from the coordinating center.

Patients’ BP level was measured by physicians or certified staff with the arm-cuff position at the heart level during rest in a seated position. The measurement was performed two times at intervals of 1–2 min by the auscultation method or by using an automatic sphygmomanometer^41^. In both groups, the BP target level of < 130/80 mmHg was applied for patients with all CKD stages, and that of < 125/75 mmHg was applied for patients with proteinuria > 1.0 g/day. The study was conducted in accordance with Ethical Guidelines for Clinical Studies (revised on December 28, 2004, of the Ministry of Health, Labor, and Welfare) and the Ethical Guidelines for Epidemiological Studies (revised on August 16, 2007, of the Ministries of Education, Culture, Sports, Science, and Technology/ Health, Labor, and Welfare).

### Patients

**Figure 4:**
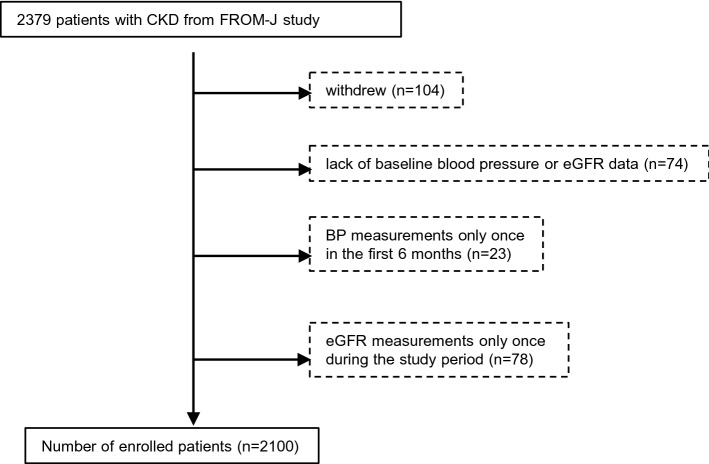
Flow chart of patients. *CKD* chronic kidney disease, *FROM-J* Frontier of Renal Outcome Modifications in Japan, *eGFR* estimated glomerular filtration rate.

In FROM-J study, a total of 2379 patients aged 40 to 74 were enrolled. Eligible participants were those who had Stage 1, 2, 4 or 5 CKD, or Stage 3 CKD with proteinuria (ratio of urinary protein/urinary creatinine ≥ 0.3 g/gCr, or proteinuria ≥ 1 +) and diabetes or Stage 3 CKD with proteinuria (ratio of urinary protein/urinary creatinine > 0.3 g/gCr or proteinuria > 1 +) and hypertension, who were under consultation with GPs. Dialysis patients, renal transplant patients, and those who did not consent were excluded from the study. Participant recruitment was from April 1, 2008 to October 19, 2008. On October 20, 2008, the local medical associations were randomly assigned at a ratio of 1:1 to group A or group B. Randomization was performed centrally by means of a com- puter-generated random-number sequence. The primary intervention study and follow-up duration lasted from October 20, 2008, to March 31, 2012. For our analyses, 104 patients who chose to withdraw, 74 patients without BP or eGFR data at baseline, 23 patients who had only one measurement of BP in the first 6 months, and 78 patients who had only one measurement of eGFR during the study period, were excluded, resulting in the final number of participants of 2100 (Fig. 4). All patients provided written informed consent.

### Outcomes

We focused on evaluating the association of BP levels with renal outcome. Given that a 40% reduction in eGFR is strongly associated with the risk of ESRD and examined as an endpoint in recent large clinical trials^42,43^, we defined renal outcome as eGFR reduction from baseline of ≥ 40% or a diagnosis of ESRD. Patients with a 40% reduction in eGFR who preserved 60 mL/min/1.73 m^2^ and more were not considered to have reached the endpoint. ESRD was defined by the initiation of dialysis or receipt of a kidney transplant.

First, we categorized baseline SBP levels into six groups: SBP < 120 mmHg, 120–129 mmHg, 130–139 mmHg, 140–149 mmHg, 150–159 mmHg, and ≥ 160 mmHg, and also categorized baseline DBP levels into four groups: DBP < 70 mmHg, 70–79 mmHg, 80–89 mmHg, and ≥ 90 mmHg. The relationship between the groups and the renal outcome were statistically analyzed. Subgroup analysis was performed based on the presence of proteinuria (the definition of proteinuria as ≥  ± by dipstick test) to assess the benefit of low BP level, which was < 130/80 mmHg at baseline, on renal outcome.

Second, we investigated the association of BP at baseline and after 1 year with the renal outcome. In patients without measurement of BP just 12 months later, the BP of the 2 months before and after was taken as the BP of 1 year later. With a target SBP level of < 130 mmHg and a target DBP level of < 80 mmHg, patients were divided into four groups based on the achievement of the target SBP and DBP levels at baseline and after 1 year, respectively. We intended to confirm whether achievement of the target BP level after 1 year was associated with reduction of the risk of the renal outcome.

Furthermore, we compared the group A with B to assess the benefit of lifestyle modification in terms of preventing CKD progression. We examined the difference between the two groups stratified by baseline eGFR and BP levels. eGFRs in this study were calculated using the following formula^44^:

$${\text{eGFR }}\left( {{\text{mL}}/{\text{min}}/{1}.{\text{73 m}}^{{2}} } \right)\, = \,{194}\, \times \,{\text{Age}}^{{ - 0.{287}}} \, \times \,{\text{Creatinine}}^{{ - {1}.0{94}}} \left( { \times \,0.{\text{739 if female}}} \right).$$

### Statistical analysis

Cumulative probability of the renal outcome was traced using the Kaplan–Meier curves and analyzed using a log-rank test. The effects of BP levels on the renal outcome were evaluated using Cox proportional hazards regression models with adjustment for age, sex, body mass index, current smoking, presence of diabetes mellitus, presence of dyslipidemia, intervention arm, presence of hyperuricemia and use of anti-hypertensive medication. We calculated the risk ratios of renal outcome for each stratified baseline BP level. A two-tailed value of P < 0.05 was defined as the statistically significant. All analyses were performed using JMP, version 15 (SAS Institute Inc., Cary, NC, USA).

## Supplementary Information

Supplementary Information.
