# Supplementary material for: Association of blood pressure and renal outcome in patients with chronic kidney disease; a post hoc analysis of FROM-J study
Source: Sci Rep. 2021 Jul 22;11:14990. doi: 10.1038/s41598-021-94467-z (PMC8298520; doi:10.1038/s41598-021-94467-z)
Supplement: Supplementary file 1 — Supplementary Information. [file 41598_2021_94467_MOESM1_ESM.pdf]

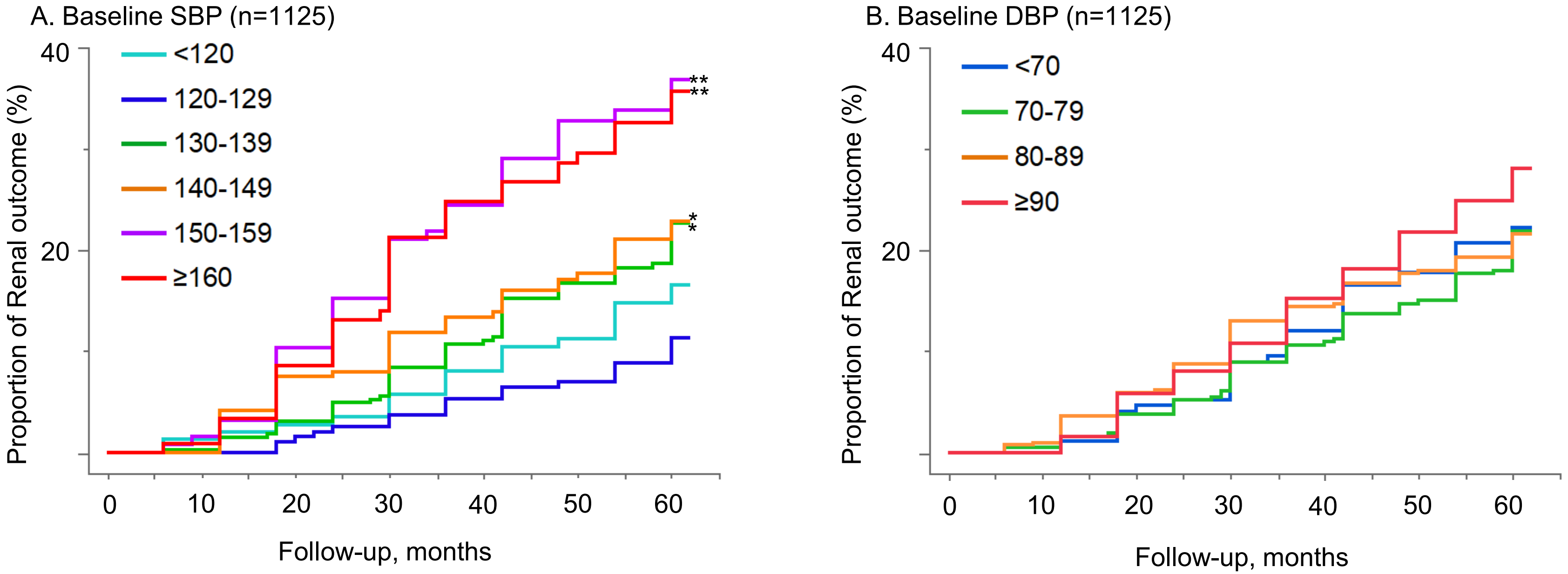

Supplemental Figure S1. Kaplan-Meier analyses of stratified analyses for renal outcome based on baseline SBP and DBP levels in patients with eGFR <60 ml/min/1.73m^2 (n=1125)

A. Analyses of baseline SBP levels; Significant increase of the renal outcome was observed in groups of SBP ≥130 mmHg compered to the group of SBP 120-129 mmHg
B. Analyses of baseline DBP levels
* p < 0.05 vs SBP 120-129mmHg (Log-rank), ** p < 0.001 vs SBP 120-129mmHg (Log-rank)

A. Analyses of SBP level at baseline and after one year (n=541)

B. Analyses of DBP level at baseline and after one year (n=541)

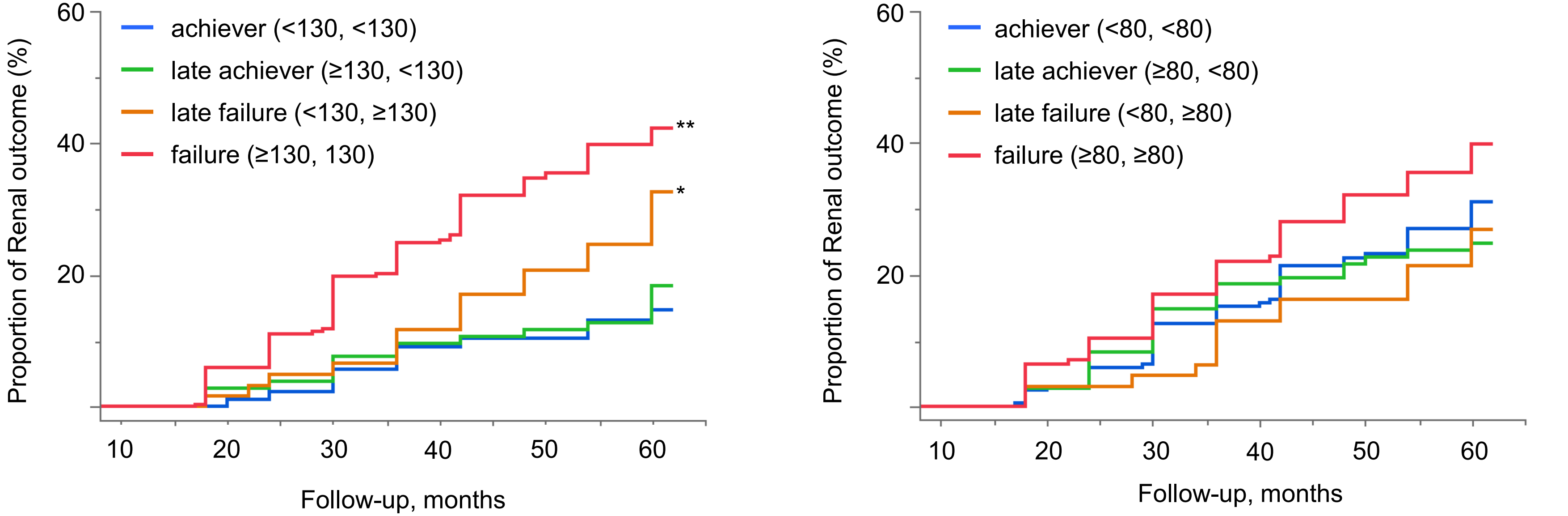

Supplemental Figure S2. Kaplan-Meier analyses of renal outcome based on management of BP levels after one year in patients with eGFR <45 ml/min/1.73m^2 (n=541)

Management of SBP to <130 mmHg or DBP to <80 mmHg after one year did not increase the risk of renal outcome in patients with eGFR <45 ml/min/1.73m^2

* p < 0.05 vs achiever (Log-rank), ** p < 0.001 vs achiever (Log-rank)

Supplemental Table S1. Baseline characteristics of patients stratified by DBP categories

Characteristics	Overall n=2100	Baseline DBP <70 n=304	70-79 n=678	80-89 n=749	90≤ n=369
Male sex, %	71.4	68.8	63.7	72.5	75.1
Age, y	62.5±8.3	65.6±7.3	63.7±8.0	62.0±8.0	58.7±8.8
BMI, kg/m^2	25.7±3.8	25.4±3.5	25.7±3.9	25.6±3.7	26.0±4.0
Abdominal girth, cm	90.0±9.9	87.8±10.2	88.5±9.7	90.7±9.2	93.4±10.5
Current smoking, %	22.5	19.6	20.6	23.5	26.6
Diabetes mellitus, %	61.1	67.8	63.5	59.0	55.6
Hypertension, %	90.8	83.5	89.5	93.2	94.6
Dyslipidemia, %	69.3	66.7	66.6	71.0	72.9
Hyperuricemia, %	38.8	39.9	35.4	41.0	39.6
**Medication**					
Anti-hypertensive, %	86.1	81.9	83.6	88.3	90.0
Use of RAS-I, %	72.8	69.1	70.8	73.7	77.8
**Laboratory values**					
Positive proteinuria, %*	80.6	78.9	77.3	81.0	87.8
Proteinuria, median (IQR), g/gCr	0.22 (0.06-0.71)	0.20 (0.07-0.65)	0.19 (0.04-0.71)	0.26 (0.07-0.71)	0.26 (0.08-0.77)
Total protein, g/dL	7.2±0.5	7.2±0.5	7.2±0.5	7.2±0.5	7.3±0.5
Albumin, g/dL	4.3±0.4	4.2±0.3	4.3±0.4	4.3±0.3	4.3±0.3
Hemoglobin, g/dL	13.8±1.8	13.1±1.7	13.6±1.7	14.0±1.7	14.4±1.8
BUN, mg/dL	19.6±8.0	21.1±8.9	19.7±8.4	19.0±7.4	19.0±7.8
Creatinine, mg/dL	1.07±0.49	1.13±0.57	1.06±0.47	1.06±0.47	1.11±0.53
eGFR, ml/min/1.73 m^2	59.1±21.9	55.8±20.8	59.2±22.6	60.2±21.7	59.3±21.7
HbA1c, %	6.2±1.2	6.0±1.1	6.1±1.1	6.1±1.2	6.2±1.3
Uric acid, mg/dL	6.2±1.2	6.1±1.7	6.0±1.4	6.2±1.7	6.3±1.5
TC, mg/dL	198±35	186±31	195±34	201±37	204±34
HDL-C, mg/dL	54±16	54±15	55±17	54±15	53±15
Non-HDL-C, mg/dL	147±37	135±32	143±36	150±38	155±37
Triglyceride, mg/dL	173±140	144±88	165±107	177±158	206±176

Unless otherwise specified, data are presented as the mean ± SD,

Abbreviations: SBP, systolic blood pressure; BMI, body mass index; CKD, chronic kidney disease; DBP, diastolic blood pressure; RAS-I, renin angiotensin system inhibitor; IQR, interquartile range; BUN, blood urea nitrogen; eGFR, estimated glomerular filtration rate; HbA1c, hemoglobin A1c; TC, total cholesterol; HDL-C, high density lipoprotein cholesterol

*Dipstick positive proteinuria of ± or higher

Supplemental Table S2. Risk of renal outcomes based on baseline SBP and DBP levels in patients with eGFR <60 ml/min/1.73m^2 (n=1125)

A. Baseline SBP

Baseline SBP	Events, %	Adjusted HR (95% CI)	*P* Value
**< 120**	14.5	1.62 (0.86-3.04)	0.131
**120-129**	10.3	1 [reference]	
**130-139**	20.2	2.23 (1.37-3.79)	0.001
**140-149**	20.9	2.27 (1.34-3.98)	0.002
**150-159**	34.1	4.02 (2.34-7.12)	< 0.001
≥ **160**	33.1	3.52 (2.06-6.19)	< 0.001

B. Baseline DBP

Baseline DBP	Events, %	Adjusted HR (95% CI)	*P* Value
**< 70**	20.0	0.97 (0.63-1.46)	0.882
**70-79**	19.6	1 [reference]	
**80-89**	20.0	1.07 (0.77-1.50)	0.670
≥ **90**	25.5	1.37 (0.92-2.01)	0.119

C. Risk ratios of renal outcomes based on baseline BP levels

Risk ratio (95%CI)	DBP <70	70≤ DBP <80	80≤ DBP <90	DBP ≥90
**SBP < 120**	1.16 (0.53-2.50)	1.24 (0.57-2.68)	1.36 (0.42-4.41)	1.96 (0.31-12.60)
**120≤ SBP <130**	1.60 (0.76-3.37)	1 [reference]	0.93 (0.42-2.08)	1.31 (0.32-5.31)
**130≤ SBP <140**	2.27 (1.10-4.72)	1.46 (0.79-2.69)	1.44 (0.80-2.63)	2.25 (1.12-4.54)
**140≤ SBP <150**	2.69 (1.22-5.92)	2.09 (1.12-3.92)	1.66 (0.90-3.05)	1.76 (0.88-3.52)
**150 ≤ SBP <160**	3.10 (1.26-7.66)	2.76 (1.34-5.67)	2.45 (1.31-4.60)	2.88 (1.54-5.37)
**SBP ≥160**	1.18 (0.17-8.09)	4.06 (2.00-8.26)	3.00 (1.53-5.89)	3.04 (1.66-5.56)

Abbreviations: SBP, systolic blood pressure; DBP, diastolic blood pressure; HR, hazard ratio; CI, Confidence interval

Hazard ratios have been adjusted for nine prespecified baseline factors (age, sex, body mass index, smoking, presence of diabetes mellitus, presence of dyslipidemia, presence of hyperuricemia, use of anti-hypertensive medication and intervention arm)

Supplemental Table S3. Risk of renal outcomes based on management of blood pressure levels after one year in patient with eGFR <45 ml/min/1.73m^2 (n=541)

A. SBP at baseline and after one year

**Group**	**SBP**		**Adjusted HR (95% CI)**	***P* Value**
	baseline	after one year		
**achiever**	<130	<130	1 [reference]	
**late achiever**	≥130	<130	1.33 (0.62-2.92)	0.463
**late failure**	<130	≥130	2.16 (0.99-4.84)	0.052
**failure**	≥130	≥130	3.40 (1.94-6.55)	< 0.001

B. DBP at baseline and after one year

**Group**	**DBP**		**Adjusted HR (95% CI)**	***P* Value**
	baseline	after one year		
**achiever**	<80	<80	1 [reference]	
**late achiever**	≥80	<80	0.80 (0.48-1.27)	0.346
**late failure**	<80	≥80	0.72 (0.37-1.29)	0.281
**failure**	≥80	≥80	1.35 (0.91-1.99)	0.133

Abbreviations: SBP, systolic blood pressure; DBP, diastolic blood pressure; HR, hazard ratio; CI, confidence interval

Hazard ratios have been adjusted for nine prespecified baseline factors (age, sex, body mass index, smoking, presence of diabetes mellitus, presence of dyslipidemia, presence of hyperuricemia, use of anti-hypertensive medication and intervention arm)
